# A Hybrid Fuzzy Decision Model for Evaluating MEMS and IC Integration Technologies

**DOI:** 10.3390/mi12030276

**Published:** 2021-03-07

**Authors:** Qian-Yo Lee, Ming-Xuan Lee, Yen-Chun Lee

**Affiliations:** 1 Department of Biomechatronic Engineering, National Chiayi University, No. 300, Syuefu Rd., Chiayi City 600355, Taiwan; yolee2300@gmail.com; 2 Graduate Institute of Electronics Engineering, National Taiwan University, No. 1, Sec. 4, Roosevelt Rd., Taipei City 10617, Taiwan; ken.lee1998@gmail.com; 3 Institute of Management of Technology, National Yang Ming Chiao Tung University, No. 1001, University Rd., Hsinchu City 300, Taiwan

**Keywords:** technology evaluation, MEMS and IC integration, MCDM, fuzzy AHP, fuzzy VIKOR

## Abstract

Integrated devices incorporating MEMS (microelectromechanical systems) with IC (integrated circuit) components have been becoming increasingly important in the era of IoT (Internet of Things). In this study, a hybrid fuzzy MCDM (multi-criteria decision making) model was proposed to effectively evaluate alternative technologies that incorporate MEMS with IC components. This model, composed of the fuzzy AHP (analytic hierarchy process) and fuzzy VIKOR (VIseKriterijumska Optimizacija I Kompromisno Resenje) methods, solves the decision problem of how best to rank MEMS and IC integration technologies in a fuzzy environment. The six important criteria and the major five alternative technologies associated with our research themes were explored through literature review and expert investigations. The priority weights of criteria were derived using fuzzy AHP. After that, fuzzy VIKOR was deployed to rank alternatives. The empirical results show that development schedule and manufacturing capability are the two most important criteria and 3D (three-dimensional) SiP (system-in-package) and monolithic SoC (system-on-chip) are the top two favored technologies. The proposed fuzzy decision model could serve as a reference for the future strategic evaluation and selection of MEMS and IC integration technologies.

## 1. Introduction

**Figure 1:**
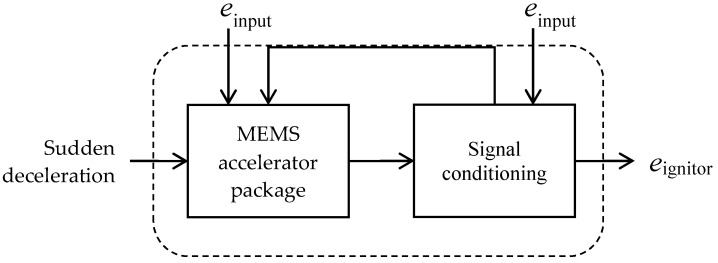
Accelerometer sensor system in an airbag system [4]. MEMS, microelectromechanical systems.

The rapid development of IoT (Internet of Things) provides great opportunities for disruptive innovations [1]. It is a remarkable fact that MEMS (microelectromechanical systems) transducers are the key enablers of IoT [2], because IoT requires a variety of input quantities that are sensed from external environments. MEMS devices act as transducers that measure or control physical, optical, or chemical quantities, such as acceleration, radiation, or fluids [3]. A typical configuration of an accelerometer sensor system in an airbag system is shown in Figure 1 [4]. The MEMS device interacts with the signal conditioning ICs to produce the amplified signal that fires the ignitor if the deceleration is high enough.

To enable MEMS sensors to function well, the electrical interfaces with the outside world need to be realized through ICs (integrated circuits) that provide electronic systems with operating intelligence [5]. ICs can definitely provide signal conditioning functions, such as analog-to-digital conversion, amplification, filtering, data processing, and communication between MEMS sensors and the outside world [6].

Until now, several viable technologies for incorporating MEMS with IC components have been either developed or are under development, but an appropriate decision making model for global semiconductor industry remains an open question. In this study, a hybrid fuzzy MCDM (multi-criteria decision making) model was proposed to facilitate the evaluation and selection of MEMS and IC integration technologies. This model, composed of fuzzy AHP (analytic hierarchy process) and fuzzy VIKOR (VIseKriterijumska Optimizacija I Kompromisno Resenje) methods, solves the decision problem of how best to rank MEMS and IC integration technologies in a fuzzy environment.

## 2. Literature Review

In this section, the literature associated with our research themes was reviewed.

### 2.1. Technology Assessment Using MCDM

In the recent decade, MCDM methods have developed rapidly and have evolved to accommodate various types of applications [7]. For example, Van de Kaa, Rezaei, Kamp, and de Winter [8] applied both crisp AHP and fuzzy AHP methods to a standardization problem for photovoltaic technological systems. Vinodh, Nagaraj, and Girubha [9] used the fuzzy VIKOR method for evaluating rapid prototyping technologies in an agile environment. Liu, You, Lu, and Chen [10] proposed a novel hybrid MCDM model for selection of healthcare waste treatment technologies. Bairagi, Dey, Sarkar, and Sanyal [11] proposed a technique for TOPSIS-based (technique for order preference by similarity to ideal solution) fuzzy MCDM approach for selecting the best robotic system. Lee and Chou [12] explored a technology selection process for evaluating 3DIC (three-dimensional integrated circuit) integration technologies. In addition, Taylan, Alamoudi, Kabli, AlJifri, Ramzi, and Herrera-Viedma [13] integrated fuzzy AHP, fuzzy VIKOR and TOPSIS methods to determine the most eligible energy systems for investment. Salimi, Noori, Bonakdari, Masoompour Samakosh, Sharifi, Hassanvand, Agharazi, and Gharabaghi [14] integrated fuzzy AHP and fuzzy VIKOR methods based on group decision making to examine the role of mass media advertising types on improving the water consumption behavior. In view of the associated literature mentioned above, MCDM methods have demonstrated their importance as optimal candidates in the decision making of emerging technologies.

### 2.2. Alternative Technologies

Several viable technologies for incorporating MEMS with IC components have been either developed or are under development, and they can basically be divided into two major solutions: (1) hybrid multi-chip solutions and (2) SoC (system-on-chip) solutions [5]. Hybrid integration of MEMS and IC components has been dominated by 2D (two-dimensional) integration approaches in which each of MEMS and IC wafers are fabricated independently [15]. These individual wafers are then separated into discrete chips and finally integrated into MCMs (multi-chip modules) [16]. Another approach for the hybrid integration of MEMS and IC components is SiP (system-in-package), which is also known as vertically stacked MCMs [17,18]. In this case, discrete chips are attached on top of each other and interconnected via wire bonding or flip-chip bonding [18,19,20]. Moreover, SoP (system-on-package) is another approach in which MEMS and IC chips are integrated with other technologies, enabling highly integrated and miniaturized systems at package levels [21,22].

SoC solutions are characterized by incorporating MEMS with IC components on the same wafers in which chip separation occurs only at or near the end of manufacturing processes [5,23]. They can be categorized into two major approaches: (1) monolithic MEMS and IC integration approaches in which MEMS and IC structures are manufactured altogether on the same wafers [24] and (2) heterogeneous MEMS and IC integration approaches in which MEMS and IC structures are premanufactured on discrete wafers and then merged onto the same substrates via wafer bonding techniques [25]. Monolithic MEMS and IC integration approaches can be further categorized into four techniques [26]: (1) monolithic MEMS and IC integration using MEMS-first processing [27], (2) monolithic MEMS and IC integration using interleaved MEMS and IC processing [28], (3) monolithic MEMS and IC integration using MEMS-last processing via bulk micromachining of IC substrates (also known as CMOS-MEMS (complementary metal-oxide-semiconductor) [29], and (4) monolithic MEMS and IC integration using MEMS-last processing via layer deposition and surface micromachining [30]. Another SoC solution based on heterogeneous MEMS and IC integration approaches can be also categorized into two techniques [31]: (1) heterogeneous MEMS and IC integration via formation during layer transfer and (2) heterogeneous MEMS and IC integration via formation after layer transfer.

## 3. Research Methods

The methodologies of fuzzy AHP and fuzzy VIKOR methods are illustrated below.

### 3.1. Fuzzy AHP Method

The AHP method, developed by Saaty [32,33], has been criticized because a decision problem can be structured in a hierarchical manner. However, AHP cannot effectively reflect the ambiguity in human thinking style [34]. To solve this problem, fuzzy AHP was thus proposed [35,36]. The procedure of fuzzy AHP is described in the following steps [12]:

Step 1  Define a problem.
Step 2  Determine important criteria.
Step 3  Establish a hierarchical structure.
Step 4  Determine linguistic variables.
Step 5  Construct fuzzy judgment matrices.

A fuzzy judgment matrix can be defined as follows:

(1) $${\tilde{A}}^{k}={\left[{\tilde{a}}_{ij}\right]}^{k}$$

$${\tilde{a}}_{ij}^{k}=\left(1,\;1,\;1\right),\;\forall \;i=j,\;\mathrm{and}\;{\tilde{a}}_{ji}^{k}=1/{\tilde{a}}_{ij}^{k},\;\forall \;i,j=1,\;2,\;\ldots ,\;n$$

where ${\tilde{A}}^{k}$ is a fuzzy judgment matrix evaluated by expert *k* (k=1,2,⋯,K), ${\tilde{a}}_{ij}^{k}$ is fuzzy assessment between criterion *i* and criterion *j* evaluated by expert *k*, and *n* is the number of criteria at the same level.

Step 6  Check consistency.

If *A* is consistent, then $\tilde{A}$ is accordingly consistent [37]. To verify whether *A* is sufficiently consistent, the maximum eigenvalue $\lambda_{\max}$ can be computed as follows:

(2) $$A\circ W=W^{\prime }=\lambda_{\max}\circ W$$

where *A* is a pairwise comparison matrix and *W* is a weight matrix.

(3) $$\lambda_{\max}=\frac{1}{n}\left(\frac{W_{1}^{\prime }}{W_{1}}+\frac{W_{2}^{\prime }}{W_{2}}+\cdots +\frac{W_{n}^{\prime }}{W_{n}}\right)$$

Saaty [33] proposed a consistency index (*CI*) to check the consistency within pairwise comparison matrices, as well as that of the entire hierarchy. The *CI* is formulated as follows:

(4) $$CI=\left(\lambda_{\max}-n\right)/\left(n-1\right)$$

where *n* is the dimension of matrix *A*.

The consistency ratio (*CR*) is accordingly defined as follows:

(5) CR=CI/RI

where *RI* is the random consistency index.

The pairwise comparison matrix *A* is considered consistent if the resulting ratio *CR* is less than 0.1.

Step 7  Integrate experts’ opinions.

In order to integrate experts’ opinions, a fuzzy synthetic judgment matrix can be obtained using the geometric mean technique [38] to compute fuzzy geometric means of each criterion [39]. Then, fuzzy weights of each criterion can be computed using the arithmetic mean technique as follows:

(6) $$\tilde{B}=\left[{\tilde{b}}_{ij}\right]\;,\;\forall \;i,j$$

(7) $${\tilde{b}}_{ij}={\left({\tilde{a}}_{ij}^{1}⊗\cdots ⊗{\tilde{a}}_{ij}^{k}⊗\cdots ⊗{\tilde{a}}_{ij}^{K}\right)}^{1/K}$$

(8) $${\tilde{r}}_{i}={\left({\tilde{b}}_{ij}^{1}⊗{\tilde{b}}_{ij}^{2}⊗\cdots ⊗{\tilde{b}}_{ij}^{n}\right)}^{1/n}$$

(9) $${\tilde{w}}_{i}={\tilde{r}}_{i}⊗{\left({\tilde{r}}_{1}⊕{\tilde{r}}_{2}⊕\cdots ⊕{\tilde{r}}_{n}\right)}^{-1}$$

where $\tilde{B}$ is a fuzzy synthetic judgment matrix of total *K* experts, ${\tilde{b}}_{ij}$ is a geometric mean of fuzzy assessment of total *K* experts, ${\tilde{r}}_{ij}$ is a geometric mean of a row of the fuzzy synthetic judgment matrix $\tilde{B}$, and ${\tilde{w}}_{i}$ is a fuzzy weight of the *i*th criterion.

Step 8  Defuzzify fuzzy weights.

Using the centroid defuzzification technique to locate BNP (best nonfuzzy performance) values [12,40], fuzzy weights of criteria can be defuzzified as crisp values.

(10) $$DF_{{\tilde{w}}_{i}}=BNP_{{\tilde{w}}_{i}}≅\frac{1}{3}\left(L_{{\tilde{w}}_{i}}+M_{{\tilde{w}}_{i}}+U_{{\tilde{w}}_{i}}\right)$$

where $L_{{\tilde{w}}_{i}}$, $M_{{\tilde{w}}_{i}}$, and $U_{{\tilde{w}}_{i}}$ represent the lower, middle, and upper values of the fuzzy weight of the *i*th criterion.

### 3.2. Fuzzy VIKOR Method

The VIKOR method helps decision-makers to determine compromise solutions for a problem and to rank and select from a set of alternatives over conflicting and incommensurable criteria for reaching ideal/aspired levels [41]. In the past few decades, an extension of VIKOR, namely fuzzy VIKOR, has been further combined with fuzzy set theory to determine compromise solutions under the fuzzy environment where both criteria and weights could be fuzzy sets [42,43]. The procedure of fuzzy VIKOR is described in the following steps [44,45,46].

Step 1  Determine a group of experts.

Let $A_{i}\;\left(i=1,2,\cdots ,m\right)$ be a be a finite set of *m* alternatives that are to be evaluated by hte *k*th expert $\left(E_{k}\;,\;k=1,2,\cdots ,K\right)$ with respect to a set of *n* criteria $\left(C_{j}\;,\;j=1,2,\cdots ,n\right)$.

Step 2  Determine linguistic variables.
Step 3  Obtain fuzzy performance rating matrices.

A typical fuzzy VIKOR questionnaire can be expressed in a matrix format as follows:

(11) $${\tilde{X}}_{k}={\left[{\tilde{x}}_{ijk}\right]}_{m\times n}$$

where ${\tilde{x}}_{ijk}$ is the fuzzy performance rating of alternative $A_{i}$ with respect to criterion $C_{j}$ evaluated by expert $E_{k}$. ${\tilde{x}}_{ijk}=\left(x_{ijk}^{l},x_{ijk}^{m},x_{ijk}^{u}\right)$ is a linguistic variable denoted by a TFN (triangular fuzzy number).

Step 4  Construct an aggregated fuzzy performance rating matrix.

An aggregated fuzzy performance rating $\tilde{D}$ can be constructed with *m* alternatives and *n* criteria as follows:

(12) $$\tilde{D}={\left[{\tilde{D}}_{ij}\right]}_{m\times n}=\begin{array}{cc} & \begin{array}{ccccc} {\tilde{C}}_{1} & \cdots & {\tilde{C}}_{j} & \cdots & {\tilde{C}}_{n} \end{array}\\ \begin{array}{c} {\tilde{A}}_{1}\\ \vdots \\ {\tilde{A}}_{i}\\ \vdots \\ {\tilde{A}}_{m} \end{array} & \left[\begin{array}{ccccc} {\tilde{x}}_{11} & \cdots & {\tilde{x}}_{1j} & \cdots & {\tilde{x}}_{1n}\\ \vdots & & \vdots & & \vdots \\ {\tilde{x}}_{i1} & \cdots & {\tilde{x}}_{ij} & \cdots & {\tilde{x}}_{in}\\ \vdots & & \vdots & & \vdots \\ {\tilde{x}}_{m1} & \cdots & {\tilde{x}}_{mj} & \cdots & {\tilde{x}}_{mn} \end{array}\right] \end{array}$$

where ${\tilde{x}}_{ij}=\left(x_{ij}^{l},x_{ij}^{m},x_{ij}^{u}\right)$ is the fuzzy performance rating of *i*th alternative with respect to *j*th criterion, and

(13) $$x_{ij}^{l}=\frac{1}{K}\overset{K}{\underset{k=1}{\sum }}x_{ijk}^{l}\;x_{ij}^{m}=\frac{1}{K}\overset{K}{\underset{k=1}{\sum }}x_{ijk}^{m}\;x_{ij}^{m}=\frac{1}{K}\overset{K}{\underset{k=1}{\sum }}x_{ijk}^{m}$$

Step 5  Determine the fuzzy best value and fuzzy worst value.

The fuzzy best value and the fuzzy worst value are determined as follows:

(14) $${\tilde{x}}_{j}^{+}=\left\{\left(\underset{i}{\max}{\tilde{x}}_{ij}|j\in B\right),\left(\underset{i}{\min}{\tilde{x}}_{ij}|j\in C\right)|\forall j=1,2,\cdots ,n\right\}$$

(15) $${\tilde{x}}_{j}^{-}=\left\{\left(\underset{i}{\min}{\tilde{x}}_{ij}|j\in B\right),\left(\underset{i}{\max}{\tilde{x}}_{ij}|j\in C\right)|\forall j=1,2,\cdots ,n\right\}$$

where ${\tilde{x}}_{j}^{+}$ is the fuzzy positive ideal solution (FPIS) and ${\tilde{x}}_{j}^{-}$ is the fuzzy negative ideal solution (FNIS) for the *j*th criterion. *B* belongs to the benefit criteria and *C* belongs to the cost criteria.

Step 6  Calculate utility and regret measures.

The VIKOR ranking indicates that the preferred alternative is proximate to the ideal solution, starting from the $L^{p}$-metric used as an aggregating function in a compromise programming method as follows:

(16) $$L_{i}^{p}={\left\{\overset{n}{\underset{j=1}{\sum }}{\left|{\tilde{w}}_{j}\frac{\left({\tilde{x}}_{j}^{+}-{\tilde{x}}_{ij}\right)}{\left({\tilde{x}}_{j}^{+}-{\tilde{x}}_{j}^{-}\right)}\right|}^{p}\right\}}^{1/p},1\leq p\leq \infty ;i=1,2,\cdots ,m$$

In the VIKOR method, $L_{i}^{p=1}$ as ${\tilde{S}}_{i}$ and $L_{i}^{p=\infty }$ as ${\tilde{R}}_{i}$ are used to formulate the ranking measure as follows:

(17) $${\tilde{S}}_{i}=\overset{n}{\underset{j=1}{\sum }}\left|{\tilde{w}}_{j}{\tilde{r}}_{ij}\right|=\overset{n}{\underset{j=1}{\sum }}\left|{\tilde{w}}_{j}\frac{\left({\tilde{x}}_{j}^{+}-{\tilde{x}}_{ij}\right)}{\left({\tilde{x}}_{j}^{+}-{\tilde{x}}_{j}^{-}\right)}\right|,\;i=1,2,\cdots ,m$$

(18) $${\tilde{R}}_{i}=\underset{j}{\max}\left|{\tilde{w}}_{j}{\tilde{r}}_{ij}\right|=\underset{j}{\max}\left|{\tilde{w}}_{j}\frac{\left({\tilde{x}}_{j}^{+}-{\tilde{x}}_{ij}\right)}{\left({\tilde{x}}_{j}^{+}-{\tilde{x}}_{j}^{-}\right)}\right|\;,i=1,2,\cdots ,m$$

where ${\tilde{S}}_{i}$ and ${\tilde{R}}_{i}$ represent the utility measure and the regret measure, respectively; ${\tilde{S}}_{i}$ is shown as the average gap for achieving the aspired level; ${\tilde{R}}_{i}$ is shown as the maximal gap for improving the priority; ${\tilde{S}}_{i}$ refers to the separation measure of $A_{i}$ from the positive-ideal solution; ${\tilde{R}}_{i}$ is the separation measure of $A_{i}$ from the negative-ideal solution; and ${\tilde{w}}_{j}$ are the fuzzy weights of criteria.

Step 7  Compute index value.

To obtain the ranking results, the index value ${\tilde{Q}}_{i}$ is computed as follows:

(19) $${\tilde{Q}}_{i}=v\frac{\left({\tilde{S}}_{i}-{\tilde{S}}^{+}\right)}{\left({\tilde{S}}^{-}-{\tilde{S}}^{+}\right)}+\left(1-v\right)\frac{\left({\tilde{R}}_{i}-{\tilde{R}}^{+}\right)}{\left({\tilde{R}}^{-}-{\tilde{R}}^{+}\right)}\;,i=1,2,\cdots ,m$$

where

(20) $${\tilde{S}}^{+}=\underset{i}{\min}{\tilde{S}}_{i}\;;\;{\tilde{S}}^{-}=\underset{i}{\max}{\tilde{S}}_{i},\;{\tilde{R}}^{+}=\underset{i}{\min}{\tilde{R}}_{i}\;;\;R^{-}=\underset{i}{\max}{\tilde{R}}_{i}$$

The indices ${\tilde{S}}^{+}$ and ${\tilde{R}}^{+}$ are related to a maximum group utility (majority rule) and a minimum individual regret of an opponent strategy, respectively. The parameter v∈[0,1] is defined as the weight for the decision-making strategy of maximum group utility, whereas (1−v) is defined as the weight for the decision-making strategy of minimum individual regret.

Step 10  Defuzzify TFNs

Using the centroid defuzzification technique to locate BNP values [12,40], the TFNs ${\tilde{S}}_{i}=\left(S_{i}^{l},S_{i}^{m},S_{i}^{u}\right)$, ${\tilde{R}}_{i}=\left(R_{i}^{l},R_{i}^{m},R_{i}^{u}\right)$, and ${\tilde{Q}}_{i}=\left(Q_{i}^{l},Q_{i}^{m},Q_{i}^{u}\right)$ can be defuzzified as the crisp values $S_{i}$, $R_{i}$, and $Q_{i}$, respectively.

(21) $$\begin{array}{c} S_{i}≅\frac{1}{3}\left(S_{i}^{l}+S_{i}^{m}+S_{i}^{u}\right)\\ R_{i}≅\frac{1}{3}\left(R_{i}^{l}+R_{i}^{m}+R_{i}^{u}\right)\\ Q_{i}≅\frac{1}{3}\left(Q_{i}^{l}+Q_{i}^{m}+Q_{i}^{u}\right) \end{array}$$

Step 11  Rank alternatives.

An index $Q_{i}$ represents the separation measure of alternative $A_{i}$ from a positive-ideal solution. The smaller the value of $Q_{i}$, the better the alternative.

Step 12  Derive a compromise solution.

A solution with a minimum $Q_{i}$ value in the ranking list is considered the optimal compromise one, if the following two conditions are satisfied:

Condition 1  Acceptable advantage

An alternative $A^{\left(1\right)}$ has an acceptable advantage if $\left(\tilde{Q}\left(A^{\left(2\right)}\right)-\tilde{Q}\left(A^{\left(1\right)}\right)\right)/\left(\tilde{Q}\left(A^{\left(n\right)}\right)-\tilde{Q}\left(A^{\left(1\right)}\right)\right)\;\geq \;1/\left(n-1\right)$, where $A^{\left(1\right)}$ is the best ranked alternative and $A^{\left(2\right)}$ is the alternative with second position in the ranking list by the measure $\tilde{Q}$. *n* is the number of alternatives.

Condition 2  Acceptable stability

An alternative $A^{\left(1\right)}$ must also be the best ranked by *S* or/and *R*. The compromise solution is stable within a decision-making process, which could be with “voting by majority rule” (when *v* > 0.5 is needed), with “consensus” (when *v* = 0.5), or with “veto” (when *v* < 0.5).

## 4. Numerical Analysis

In this section, an empirical work is definitely conducted and illustrated step-by-step according to the proposed hybrid fuzzy decision model.

Step 1  Establish the hierarchical model.

**Figure 2:**
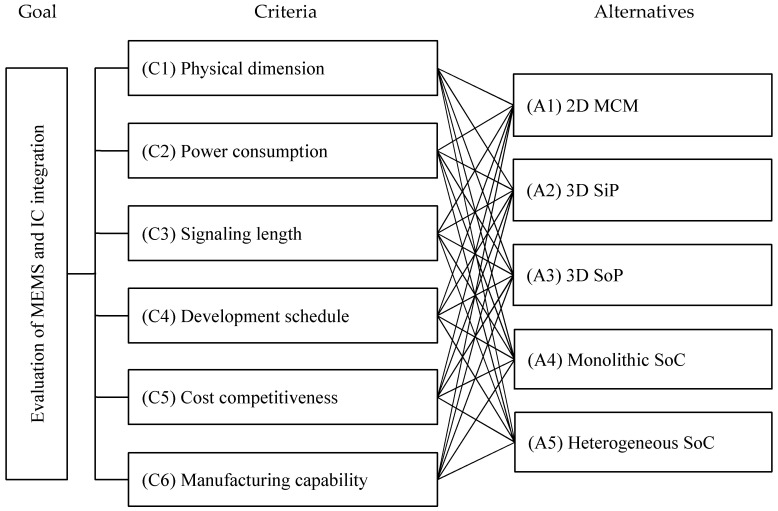
The hierarchical model for evaluating MEMS and IC (integrated circuit) integration technologies. MCM, multi-chip module; SiP, system-in-package; SoP, system-on-package; SoC, system-on-chip.

This study explored the six important criteria and concluded on the major five alternative technologies associated with our research themes through literature review and expert investigations. The hierarchical model was thus established as shown in Figure 2.

Step 2  Derive the fuzzy preference weights of the criteria.

(1) Design a questionnaire for the data collection.

A typical AHP questionnaire used a nine-point rating scale (see Table 1) to represent the relative importance of each criterion.

(2) Generate the fuzzy judgment matrices.

The fuzzy judgment matrices for the criteria from twelve experts were generated by Equation (1). Table 2 shows an individual fuzzy judgment matrix of expert 1.

(3) Check the consistency.

Using Equations (4) and (5), the *CI* value is 0.022 and the *CR* value is 0.018 (less than 0.1), indicating the consistency of the collected data in the questionnaires and the robustness of fuzzy judgment matrices.

(4) Integrate the experts’ opinions.

Using Equations (6) and (7) to compute the fuzzy geometric means of each criterion, the fuzzy synthetic judgment matrix was thus obtained as shown in Table 3.

After that, using Equations (8) and (9), the fuzzy preference weight of each criterion was obtained as shown in Table 4.

(5) Defuzzify the fuzzy weights.

Equation (10) was used to compute the BNP value of the fuzzy preference weight of each criterion. Table 5 shows the empirical results in which C4 (development schedule) and C6 (manufacturing capability) are the two most important criteria.

Step 3  Rate the alternatives.

(1) Design a questionnaire for the data collection.
(2) Generate the fuzzy performance rating matrices.

Using Equation (11), the fuzzy performance rating matrices for the alternatives with respect to the criteria were generated (see Table 6).

(3) Integrate the experts’ opinions.

Using Equations (12) and (13), the fuzzy synthetic performance rating matrix for the alternatives with respect to the criteria was thus obtained as shown in Table 7.

(4) Determine the fuzzy best values and fuzzy worst values.

Using Equations (14) and (15), the FPIS and NPIS reference points for each criterion were determined as shown in Table 8.

(5) Obtain the weighted fuzzy synthetic normalized rating matrix.

Using the equation $\left|{\tilde{w}}_{j}{\tilde{r}}_{ij}\right|=\left|{\tilde{w}}_{j}\frac{\left({\tilde{x}}_{j}^{+}-{\tilde{x}}_{ij}\right)}{\left({\tilde{x}}_{j}^{+}-{\tilde{x}}_{j}^{-}\right)}\right|,\;i=1,2,\cdots ,m$, the weighted fuzzy synthetic normalized rating matrix was obtained as shown in Table 9.

(6) Rank the alternatives.

The separation values ${\tilde{S}}_{i}$ and ${\tilde{R}}_{i}$ were calculated through Equations (17) and (18), respectively, and the index value ${\tilde{Q}}_{i}$ was computed based on Equations (19) and (20). The BNP values of ${\tilde{Q}}_{i}$ were then obtained through Equation (21). The defuzzified crisp value $Q_{i}$ represents the separation measure of alternative $A_{i}$ from a positive-ideal solution. Table 10 shows that A2 (3D SiP) and A4 (monolithic SoC) are the top two favored technologies.

## 5. Discussion

The two most important criteria were reviewed in Section 5.1. While 3D SiP and monolithic SoC were selected as the two most favored technologies, the rationale for them is also discussed in Section 5.2.

### 5.1. Rationale for the Two Most Important Criteria

In view of the fuzzy AHP results shown in Table 5, the two most important criteria are development schedule (0.260) and manufacturing capability (0.237). The development schedule indicates the roadmap of technology development through the expense of R&D (research and development) resources. Semiconductor firms would gain more competitive advantages if the development schedule of new technologies can be further reduced. Manufacturing capability refers to the technical and physical limitations of semiconductor firms. Higher manufacturing capability always leads to higher manufacturing efficiency and yield. Hence, the development schedule and manufacturing capability are the two key factors that should be considered first while evaluating the optimal alternative technology for incorporating MEMS with IC components.

### 5.2. Rationale for the Top Two Preferable Technologies

In view of the fuzzy VIKOR results shown in Table 10, 3D SiP is of primary interest to semiconductor firms among the five alternatives. The key advantages of 3D SiP technology are its higher integration densities, shorter signaling lengths, and smaller package footprints in comparison with 2D MCM. This method yields very compact packages (i.e., benefiting by physical dimensions and signaling length) [19,47] and has been employed in a number of commercial products (i.e., benefiting by development schedule and manufacturing capability) [48,49]. 3D SoP is another technology that enables a highly integrated and miniaturized system at the package level, but it went a step beyond 3D SiP by integrating thin-film components on a package substrate (i.e., suffering from development schedule and manufacturing capability) [50].

The second-ranked technology is monolithic SoC. The CMOS-MEMS technique provides advantages such as that it can be implemented using existing IC infrastructure and MEMS components can be formed in completed wafers using cost-effective processing steps (i.e., benefiting by development schedule and cost competitiveness) [5]. The heterogeneous SoC integration approach can be also supported by an existing foundry structure, but it often requires accurate substrate-to-substrate or wafer-to-wafer alignment during bonding, reliable electrical interconnections, and/or even a greater number of manufacturing steps (i.e., suffering from development schedule and manufacturing capability).

While comparing CMOS-MEMS with 3D SiP, 3D SiP has greater performance ratings than CMOS-MEMS in terms of the major two criteria—development schedule and manufacturing capability. On the other hand, CMOS-MEMS has greater performance ratings than 3D SiP in terms of the major two criteria—physical dimension and signaling length.

## 6. Conclusions

MEMS sensors are now prevalent in the era of IoT. The global semiconductor industry expects a higher integration of mechanical structures with electronics that can be manufactured by CMOS technologies as usual. A variety of microfabrication and integration approaches have been attempted, but each has distinguishing features. This study successfully proposed a hybrid fuzzy MCDM model that effectively facilitates the evaluation and selection of MEMS and IC integration technologies in a fuzzy environment. The six important criteria and the major five alternative technologies associated with our research themes were first explored through literature review and expert investigations. The priority weights of criteria were then derived using fuzzy AHP, and the two most important criteria are development schedule and manufacturing capability. After that, fuzzy VIKOR was deployed to rate the alternatives, and 3D SiP and monolithic SoC are the top two favored technologies. The proposed fuzzy decision model could serve as a reference for the future strategic evaluation and selection of MEMS and IC integration technologies.

## Figures and Tables

**Table 1:** Linguistic variables and corresponding TFNs (triangular fuzzy numbers).

TFNs	Linguistic Variables	Scale of TFNs
$\tilde{9}$	Extremely more important	(8, 9, 10)
$\tilde{8}$	Intermediate	(7, 8, 9)
$\tilde{7}$	Very strongly more important	(6, 7, 8)
$\tilde{6}$	Intermediate	(5, 6, 7)
$\tilde{5}$	Strongly more important	(4, 5, 6)
$\tilde{4}$	Intermediate	(3, 4, 5)
$\tilde{3}$	Moderately more important	(2, 3, 4)
$\tilde{2}$	Intermediate	(1, 2, 3)
$\tilde{1}$	Equally important	(1, 1, 1)

**Table 2:** Individual fuzzy judgment matrix of expert 1.

Criteria	C1	C2	C3	C4	C5	C6
C1	(1, 1, 1)	(1, 2, 3)	(1, 2, 3)	(1, 1, 1)	(1, 2, 3)	(1, 1, 1)
C2	(1/3, 1/2, 1)	(1, 1, 1)	(1, 1, 1)	(1/3, 1/2, 1)	(1, 1, 1)	(1/3, 1/2, 1)
C3	(1/3, 1/2, 1)	(1, 1, 1)	(1, 1, 1)	(1/4, 1/3, 1/2)	(1/3, 1/2, 1)	(1/3,1/2, 1)
C4	(1, 1, 1)	(1, 2, 3)	(2, 3, 4)	(1, 1, 1)	(1, 2, 3)	(1, 1, 1)
C5	(1/3, 1/2, 1)	(1, 1, 1)	(1, 2, 3)	(1/3, 1/2, 1)	(1, 1, 1)	(1/3, 1/2, 1)
C6	(1, 1, 1)	(1, 2, 3)	(1, 2, 3)	(1, 1, 1)	(1, 2, 3)	(1, 1, 1)

*λ*_max_ = 6.056; *CI* = 0.022; *CR* = 0.018 ≤ 0.1

**Table 3:** The fuzzy synthetic judgment matrix.

Criteria	C1	C2	C3	C4	C5	C6
C1	(1, 1, 1)	(1.26, 1.93, 2.51)	(1.26, 2.29, 3.3)	(0.69, 0.79, 1)	(1, 1.33, 1.58)	(0.69, 0.79, 1)
C2	(0.4, 0.52, 0.79)	(1, 1, 1)	(1.26, 1.72, 2.09)	(0.4, 0.52, 0.79)	(0.76, 0.84, 1)	(0.3, 0.44, 0.79)
C3	(0.3, 0.44, 0.79)	(0.48, 0.58, 0.79)	(1, 1, 1)	(0.27, 0.37, 0.59)	(0.4, 0.52, 0.79)	(0.4, 0.52, 0.79)
C4	(1, 1.26, 1.44)	(1.26, 1.93, 2.51)	(1.68, 2.71, 3.72)	(1, 1, 1)	(1.26, 1.93, 2.51)	(1, 1.26, 1.44)
C5	(0.63, 0.75, 1)	(1, 1.19, 1.32)	(1.26, 1.93, 2.51)	(0.4, 0.52, 0.79)	(1, 1, 1)	(0.4, 0.52, 0.79)
C6	(1, 1.26, 1.44)	(1.26, 2.29, 3.3)	(1.26, 1.93, 2.51)	(0.69, 0.79, 1)	(1.26, 1.93, 2.51)	(1, 1, 1)

**Table 4:** The fuzzy preference weights of the criteria.

Fuzzy Weights	*L*	*M*	*U*
${\tilde{w}}_{1}$	0.118	0.194	0.312
${\tilde{w}}_{2}$	0.074	0.116	0.205
${\tilde{w}}_{3}$	0.053	0.084	0.160
${\tilde{w}}_{4}$	0.145	0.247	0.388
${\tilde{w}}_{5}$	0.087	0.137	0.229
${\tilde{w}}_{6}$	0.130	0.222	0.358

**Table 5:** Best nonfuzzy performance (BNP) values of the criteria.

Criteria	BNP Values	Rank
C1 (Physical dimension)	0.208	**3**
C2 (Power consumption)	0.131	**5**
C3 (Signaling length)	0.099	**6**
C4 (Development schedule)	0.260	**1**
C5 (Cost competitiveness)	0.151	**4**
C6 (Manufacturing capability)	0.237	**2**

**Table 6:** Individual fuzzy performance rating matrix of expert 1.

Criteria	A1	A2	A3	A4	A5
C1	(2, 3, 4)	(7, 8, 9)	(8, 9, 10)	(7, 8, 9)	(8, 9, 10)
C2	(3, 4, 5)	(7, 8, 9)	(7, 8, 9)	(6, 7, 8)	(6, 7, 8)
C3	(2, 3, 4)	(5, 6, 7)	(5, 6, 7)	(6, 7, 8)	(8, 9, 10)
C4	(8, 9, 10)	(7, 8, 9)	(3, 4, 5)	(5, 6, 7)	(2, 3, 4)
C5	(7, 8, 9)	(6, 7, 8)	(4, 5, 6)	(5, 6, 7)	(5, 6, 7)
C6	(7, 8, 9)	(7, 8, 9)	(3, 4, 5)	(6, 7, 8)	(2, 3, 4)

**Table 7:** The fuzzy synthetic performance rating matrix.

Criteria	A1	A2	A3	A4	A5
C1	(2.42, 3.42, 4.42)	(6.67, 7.67, 8.67)	(6.75, 7.75, 8.75)	(7.17, 8.17, 9.17)	(7.25, 8.25, 9.25)
C2	(1.75, 2.75, 3.75)	(6.67, 7.67, 8.67)	(6.67, 7.67, 8.67)	(6.17, 7.17, 8.17)	(5.67, 6.67, 7.67)
C3	(1.75, 2.75, 3.75)	(4.83, 5.83, 6.83)	(5.33, 6.33, 7.33)	(6.33, 7.33, 8.33)	(7.67, 8.67, 9.67)
C4	(7.58, 8.58, 9.58)	(7.17, 8.17, 9.17)	(3.67, 4.67, 5.67)	(5.83, 6.83, 7.83)	(2.08, 3.08, 4.08)
C5	(7.33, 8.33, 9.33)	(6.83, 7.83, 8.83)	(5.33, 6.33, 7.33)	(6.33, 7.33, 8.33)	(5.83, 6.83, 7.83)
C6	(7.33, 8.33, 9.33)	(7.25, 8.25, 9.25)	(3.83, 4.83, 5.83)	(5.92, 6.92, 7.92)	(2.25, 3.25, 4.25)

**Table 8:** The FPIS (fuzzy positive ideal solution) and FNIS (fuzzy negative ideal solution).

Criteria	FPIS ${\tilde{x}}_{j}^{+}$	FNIS ${\tilde{x}}_{j}^{-}$
C1	(7.25, 8.25, 9.25)	(2.42, 3.42, 4.42)
C2	(6.67, 7.67, 8.67)	(1.75, 2.75, 3.75)
C3	(7.67, 8.67, 9.67)	(1.75, 2.75, 3.75)
C4	(7.58, 8.58, 9.58)	(2.08, 3.08, 4.08)
C5	(7.33, 8.33, 9.33)	(5.33, 6.33, 7.33)
C6	(7.33, 8.33, 9.33)	(2.25, 3.25, 4.25)

**Table 9:** The weighted fuzzy synthetic normalized rating matrix.

Criteria	A1	A2	A3	A4	A5
C1	(0.12, 0.19, 0.31)	(0.01, 0.02, 0.04)	(0.01, 0.02, 0.03)	(0, 0, 0.01)	(0, 0, 0)
C2	(0.07, 0.12, 0.2)	(0, 0, 0)	(0, 0, 0)	(0.01, 0.01, 0.02)	(0.01, 0.02, 0.04)
C3	(0.05, 0.08, 0.16)	(0.03, 0.04, 0.08)	(0.02, 0.03, 0.06)	(0.01, 0.02, 0.04)	(0, 0, 0)
C4	(0, 0, 0)	(0.01, 0.02, 0.03)	(0.1, 0.18, 0.28)	(0.05, 0.08, 0.12)	(0.14, 0.25, 0.39)
C5	(0, 0, 0)	(0.02, 0.03, 0.06)	(0.09, 0.14, 0.23)	(0.04, 0.07, 0.11)	(0.07, 0.1, 0.17)
C6	(0, 0, 0)	(0, 0, 0.01)	(0.09, 0.15, 0.25)	(0.04, 0.06, 0.1)	(0.13, 0.22, 0.36)

**Table 10:** Performance ratings of the alternatives. MCM, multi-chip module; SiP, system-in-package; SoP, system-on-package; SoC, system-on-chip; BNP, best nonfuzzy performance.

Alternatives	${\tilde{S}}_{i}$	${\tilde{R}}_{i}$	${\tilde{Q}}_{i}$	BNP of ${\tilde{Q}}_{i}$	Rank
A1 (2D MCM)	(0.24, 0.39, 0.68)	(0.12, 0.19, 0.31)	(0.26, 0.66, 1.82)	0.91	**3**
A2 (3D SiP)	(0.07, 0.12, 0.21)	(0.03, 0.04, 0.08)	(0, 0, 0)	0.00	**1**
A3 (3D SoP)	(0.31, 0.52, 0.85)	(0.10, 0.18, 0.28)	(0.28, 0.75, 1.98)	1.00	**4**
A4 (Monolithic SoC)	(0.15, 0.24, 0.40)	(0.05, 0.08, 0.12)	(0.08, 0.22, 0.54)	0.28	**2**
A5 (Heterogeneous SoC)	(0.36, 0.60, 0.96)	(0.14, 0.25, 0.39)	(0.38, 1.00, 2.64)	1.34	**5**
